# BMAL1 Drives Cisplatin Resistance in Non‐Small Cell Lung Cancer Via Lactate‐MRP1 Signaling Pathway

**DOI:** 10.1111/1759-7714.70279

**Published:** 2026-04-24

**Authors:** Zixin Shi, Ziniu Qin, Chuantao Chen, Xiaolin Yang, Yunhan Hu, Xiaohui Meng, Yuxiang Cao, Xiang Tao, Zhijian Zhang, Tiancheng Xie, Huijun Wei, Zhihao Wu

**Affiliations:** ^1^ Research Laboratory of Tumor Microenvironment Wannan Medical University Wuhu China; ^2^ School of Anesthesiology, Wannan Medical University Wuhu China; ^3^ School of Pharmacy, Wannan Medical University Wuhu China; ^4^ School of Medical Imageology, Wannan Medical University Wuhu China; ^5^ School of Clinical Medicine, Wannan Medical University Wuhu China; ^6^ Anhui Province Key Laboratory of Basic Research and Transformation of Age‐Related Diseases Wannan Medical University Wuhu China; ^7^ School of Laboratory Medicine, Wannan Medical University Wuhu China; ^8^ Anhui Province Key Laboratory of Non‐Coding RNA Basic and Clinical Transformation, Anhui Innovative Center for Drug Basic Research of Metabolic Diseases Wannan Medical University Wuhu China

**Keywords:** BMAL1, cisplatin resistance, lactate, MRP, NSCLC

## Abstract

Lung cancer, the leading cause of cancer‐related mortality, faces significant therapeutic challenges due to chemoresistance. While metabolic reprogramming and circadian disruptions are implicated in tumor progression, their interplay in driving resistance remains unclear. This study identifies BMAL1, a core circadian regulator, as a key driver and potential initiator of cisplatin resistance in non‐small cell lung cancer (NSCLC) through metabolic and oxidative stress pathways. We demonstrate that BMAL1 upregulates multidrug resistance protein MRP1 via HIF‐1α‐driven glycolysis, amplifying lactate production. Lactate activates the TAZ/c‐Jun/Snail complex to increase MRP1 expression, establishing a feedforward loop that sustains chemoresistance. Furthermore, cisplatin and etoposide induce BMAL1 expression through AKT signaling in response to oxidative stress, creating a self‐reinforcing resistance mechanism. Critically, targeting AKT or MRP1 reverses BMAL1‐mediated resistance. These findings reveal BMAL1 as a metabolic orchestrator linking circadian dysfunction to chemoresistance and propose actionable strategies—such as AKT inhibition or chronotherapy—to circumvent therapeutic failure. This work underscores the necessity of targeting circadian‐metabolic crosstalk to improve outcomes in NSCLC.

## Graphical abstract

The drug cisplatin induces BMAL1 expression through AKT signaling in response to cisplatin‐induced oxidative stress. BMAL1 activates HIF‐1α‐mediated metabolic reprogramming and lactate production, which in turn activates the TAZ/c‐Jun/Snail complex to upregulate drug efflux pump MRP1, initiating and sustaining chemoresistance.

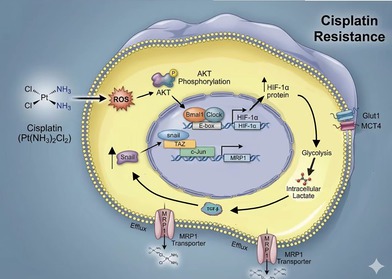

**Abbreviations**
ABCC: ATP‐binding cassette subfamily C
AKT: protein kinase B
ANOVA: analysis of variance
ARNTL: aryl hydrocarbon receptor nuclear translocator‐like
BMAL1: brain and Muscle Arnt‐Like 1
cDNA: complementary DNA
Cry: cryptochrome
DDP: cisplatin
DMEM: Dulbecco's Modified Eagle Medium
DMSO: dimethyl sulfoxide
DOX: doxorubicin
ERK: extracellular regulated protein kinases
GLUT1: glucose transporter‐1
HIF‐1α: hypoxia‐inducible factor alpha
HK2: hexokinase2
JNK: c‐Jun N‐terminal kinase
LDHA: lactate dehydrogenase A
LUAD: lung adenocarcinoma
MCT4: monocarboxylate transporter 4
MRP: multidrug‐resistance protein
MTT: 3‐(4; 5‐dimethylthiazole‐2‐yl)‐2,5‐diphenyltetrazolium bromide
NPAS2: neuronal PAS domain protein 2
NSCLC: non‐small cell lung cancer
p‐AKT: phospho‐AKT
PBS: phosphate‐buffered saline
PCR: polymerase chain reaction
PD‐L1: programmed cell death receptor‐1
Per: period
PKM2: pyruvate kinase isozyme typeM2
PTX: paclitaxel
RISC: RNA‐induced silencing complex
SD: standard deviation
siRNA: small interfering RNA
TAZ: tafazzin
TCGA: The Cancer Genome Atlas
TGF‐β1: transforming growth factor‐beta 1
TME: tumor microenvironment
TTFLs: transcriptional‐translational feedback loops
YAP: yes‐associated protein

## 1 Introduction

Lung cancer remains the leading cause of cancer‐related deaths worldwide. While targeted therapies and immunotherapies have improved survival outcomes in a subset of non‐small cell lung cancer (NSCLC) patients, significant limitations persist. Approximately 60% of NSCLC patients lack actionable genetic targets and PD‐L1 immunotherapy achieves clinical responses in only 20% of cases [1, 2, 3]. Consequently, systemic chemotherapy remains a cornerstone of NSCLC treatment. However, the development of tumor cell resistance severely compromises chemotherapeutic efficacy, directly impairing patient survival and prognosis. Thus, unraveling the molecular mechanisms underlying chemoresistance is imperative to advance therapeutic strategies.

Current research explores diverse mechanisms of drug resistance, including DNA repair defects, miRNA dysregulation and tumor microenvironment (TME) alterations [4]. Notably, metabolic reprogramming—a hallmark of cancer—plays a pivotal role in resistance [5, 6]. Tumor cells preferentially utilize glycolysis over oxidative phosphorylation (the Warburg effect), resulting in excessive lactate production. Lactate accumulation in the TME (up to 40‐fold higher than normal) correlates strongly with metastasis and poor prognosis [7]. While lactate is implicated in immunosuppression and angiogenesis [8, 9], its direct contribution to chemoresistance remains poorly defined. Furthermore, whether chemotherapy itself triggers metabolic adaptations that drive resistance is an unresolved question demanding systematic investigation.

Circadian rhythms are a fundamental characteristic of biology that help organisms synchronize their physiology and behavior with the day–night cycle caused by the Earth's rotation [10, 11]. Circadian rhythms are generated by cell‐autonomous transcriptional‐translational feedback loops (TTFLs). In mammals, the core loop involves CLOCK and BMAL1 proteins forming a heterodimer that binds E‐box promoters, activating transcription of Period (Per) and Cryptochrome (Cry) genes. PER and CRY proteins accumulate, dimerize and translocate to the nucleus to inhibit CLOCK‐BMAL1, repressing their own transcription. There is growing evidence that the circadian rhythms that control metabolism, DNA repair and cell division are significant modulators of tumorigenesis and response to treatment [10, 12]. Among core circadian genes, BMAL1 (Brain and Muscle Arnt‐Like 1) has garnered attention for its dual roles in maintaining circadian homeostasis and influencing tumor metabolism, proliferation and apoptosis [12]. Intriguingly, circadian disruptions are linked to altered drug efficacy, yet how BMAL1 intersects with metabolic pathways to mediate chemoresistance is unknown. This gap underscores the need to explore BMAL1's role in adaptive resistance mechanisms.

Our prior work revealed that the chemotherapeutic agent etoposide induces metabolic shifts toward glycolysis and lactate secretion in NSCLC cells. Building on this, we investigate BMAL1's mechanistic role in chemoresistance. Here, we demonstrate that BMAL1 activates HIF‐1α‐mediated metabolic reprogramming, amplifying glycolysis and lactate production. This cascade upregulates MRP1 (a multidrug resistance protein), establishing a feedback loop that initiates and sustains adaptive resistance. These findings not only elucidate BMAL1's role in resistance but also identify lactate‐MRP1 signaling as a targetable axis to overcome chemotherapeutic failure in NSCLC.

## 2 Materials and Methods

### 2.1 Cell Culture, Antibodies, Reagents and Plasmids

H1299 (NSCLC cell), A549 (NSCLC cell) and 293 T (human embryonic kidney) cells were purchased from National Collection of Authenticated Cell Cultures (Shanghai, China) and cultured with Dulbecco's Modified Eagle Medium (DMEM; GibcoThermo Fisher Scientific, Waltham, MA, USA) supplemented with 10% fetal bovine serum (GibcoBRL, Grand Island, NY, USA) at 37°C in a humidified atmosphere containing 5% CO_2_. Anti‐MRP1 (K003560P), anti‐HK2 (K001797P), anti‐HIF‐1α (K000487P) were obtained from Solarbio (Beijing, China). Anti‐snail (3895 s), anti‐YAP/TAZ (8418 s), anti‐AKT (9272 s) were all obtained from Cell Signaling Technology (Danvers, MA, USA). Anti‐β‐actin (A1978) was obtained from Sigma‐Aldrich (St. Louis, USA), anti‐C‐Jun (ET1608‐3) was purchased from HuaBio (Hangzhou, China), anti‐BMAL1(ABP50117) was obtained from Abbkine (Atlanta, USA), anti‐P‐AKT (4060 T) was obtained from Sigma (Vitoria, BC, Canada). Anti‐GLUT1 (A6982), anti‐SLC16A3 (MCT4) (A27772) were all purchased from Abclonal (Wuhan, China). The materials used in this study included anticancer drugs, such as etoposide (H20143143, Hainan, China), cisplatin (Cat. No:ST1164‐10 mg), Paclitaxel (SL Pharm, Beijing, China) and Doxorubicin Hydrochloride (Main Luck, Shenzhen, China), as well as reagents, including LY294002 (Cat.: 440202‐5MG), sodium citrate (Sigma, Shanghai, China, S4641‐25 g), LY2157299 (MedChamExpress, Monmouth Junction, NJ, USA, HY‐13226), 3‐(4,5‐dimethylthiazol‐2‐yl)‐2,5‐diphenyl tetrazolium bromide (MTT, Solarbio, Beijing, China, M8180) and PBS (Beyotime, Shanghai, China, C0221D).

### 2.2 Western Blot

The cells were scraped and treated with heated Laemmli sample buffer (S3401, Sigma, Victoria, BC, Canada). The homogenate was separated by SDS‐PAGE and transferred to nitrocellulose membrane (GE Healthcare, Piscataway, NJ, USA). Following a 1‐h incubation period with 5% milk and each antibody's detection, the signals were scanned using the Chemiluminescence system AI680 (Protein Simple, San Jose, CA, USA).

### 2.3 Cloning and DNA Construction

The BMAL1 promoter was amplified and inserted into the Hind III and Kpn I sites of the pGL3‐BASIC luciferase reporter vector (Promega, Madison, WI, USA). Site‐directed mutagenesis was performed using the overlapping PCR extension method, with the longest BMAL1 promoter fragment as the template to introduce specific point mutations. The primer sequences are shown below:

BMAL1 forward: 5′‐CGGGGTACCGATGGATAACGAAAAAGAAGACGCTG‐3′.

BMAL1 reverse: 5′‐CCCAAGCTTCTAACCTACTTTCCGACCAATCCGCT‐3′.

### 2.4 Dual Luciferase Reporter Assays

H1299 and A549 cells were seeded in 12‐well plates. When the cell density reached 80%, the pCMV6 plasmid containing Renilla luciferase gene, as an internal control, was co‐transfected with the MRP1 or HIF‐1a promoter luciferase reporter plasmid into the cells. An internal control for transcriptional activity was provided to ensure that the test results were not affected by variations in experimental conditions. After 48 h, the cells were lysed and both firefly luciferase and Renilla luciferase activities were measured using the dual luciferase reporter assay system. Data from three independent experiments were expressed as mean ± standard deviation (SD).

### 2.5 Cell Viability Assay

After seeding H1299 and A549 cells into 48 or 96‐well plates, where their cell density had increased to 80%, they were treated with varying drug concentrations for the same amount of time. The cells were then rinsed with PBS, treated with MTT for 4 h, DMSO was added, shaken for 15 min, formazan crystals were dissolved and absorbance was measured at 570 nm using BioTek (BioTek, Winooski, VT) microplate reader to assess cell viability. The data were then plotted using Graphpad Prism software.

### 2.6 Plasmid Transfection

Transfection was performed when the cells reached 70%–80% confluency. The medium was replaced 30 min before transfection. Plasmids and PolyJet DNA transfection reagent (Signa‐Gen Laboratories, Gaithersburg, MD, USA) were diluted in DMEM at a ratio of 1:3, thoroughly mixed to ensure complete formation of PolyJet/DNA complexes (with an incubation time of ≤ 25 min) and then added to the cells. Subsequently, the cells were incubated for 8 h to maximize transfection efficiency.

### 2.7 Small Interfering RNA (siRNA) Transfection

When the cells reached 30%–50% confluency, transfection was performed using 1 μL of GenMute siRNA Transfection Reagent (SignaGen Laboratories) and 10 nM siRNA (Table 1), followed by an 8‐h incubation. The procedure was conducted under nuclease‐free conditions. The GenMute reagent encapsulated the siRNA and formed complexes with proteins to assemble the RNA‐induced silencing complex (RISC), thereby enabling gene knockdown.

**TABLE 1:** Sequence of siRNA.

Gene	Genebank accession number	Target sequence (5′–3′)
siHIF‐1α	NM_001530	CCAGCAGACUCAAAUACAATT
siBMAL1	NR_110034	CCGAGGGAAGAUCCUCUUUTT
siABCC1	NM_004996	GAUGACACCUCUCAACAAAdTdT

### 2.8 Annexin V‐FITC/PI Double Staining Flow Cytometry

Cells were seeded into six‐well plates and cultured until they reached approximately 85% confluency. Following 12 h of serum starvation, cisplatin was added and the cells were treated for an additional 4 h. The cells were then washed and cultured for an additional 44 h before samples were prepared according to the protocol provided by the BD Pharmingen FITC Annexin V Apoptosis Detection Kit (556 547; Becton Dickinson, USA). The cell suspension was filtered through a 0.22 μm membrane into flow cytometry tubes and analyzed using Cytoflex (BGC5020, BECKMAN COULTER, Brea, USA).

### 2.9 Hoechst 33342 Efflux Assay

Cells were seeded in six‐well plates and divided into experimental groups with or without verapamil inhibitor (10 μM). After 1 h of incubation, the medium was replaced with fresh medium containing Hoechst 33342 dye (10 μg/mL). The cells were then incubated at 37°C for 10 min and fluorescence intensity was quantified using BioTek citation 5 (BioTek, Winooski, VT).

### 2.10 Glucose Uptake Assay

The cells were seeded and cultured on a 12‐well plate until the cell density reached approximately 90%. Subsequently, in accordance with the manufacturer's instructions, the Glucose Uptake‐Glo Assay (Promega, CA, USA) was employed. A solution containing cellular components was acquired by adhering to the provided experimental procedures. The solution was incubated at room temperature for 1 h. The signal integration time was set on the GloMax Navigator Microplate Luminometer (Promega, CA, USA) and the luminescent signal was then recorded.

### 2.11 Lactate Assay

The supernatants of the treated or untreated cells were added to the 96‐well plates. Then, the protocol of the Lactate Assay Kit (BioVision, Milpitas, CA, USA) was followed. Subsequently, the cells were incubated at room temperature in the dark for 30 min. The absorbance was measured at 570 nm using BioTek (BioTek, Winooski, VT) microplate reader and the concentration of lactate was determined by using the Lactate Standard Curve.

### 2.12 Statistical Analysis

Data were analyzed using SPSS 13.0 statistical software (SPSS Inc., Chicago, IL, USA) through one‐way analysis of variance (ANOVA). Results are presented as the mean ± SD of three independent experiments. A two‐tailed test was applied and statistical significance was defined as a *p*‐value < 0.05.

## 3 Results

### 3.1 BMAL1 Promotes the Resistance of Lung Cancer Cells to Cisplatin via Regulating MRP1 Expression

Disruption of circadian rhythms has been shown to promote tumorigenesis and cancer progression. Given this connection, we sought to explore whether circadian rhythm disruption might influence the resistance of cancer cells to chemotherapy drugs. To investigate this, we employed overexpression and knockdown techniques to modulate the expression of the key circadian gene BMAL1 in lung cancer cell lines and assessed its effect on initiation of resistance to cisplatin (DDP) through cell viability experiments.

**FIGURE 1:**
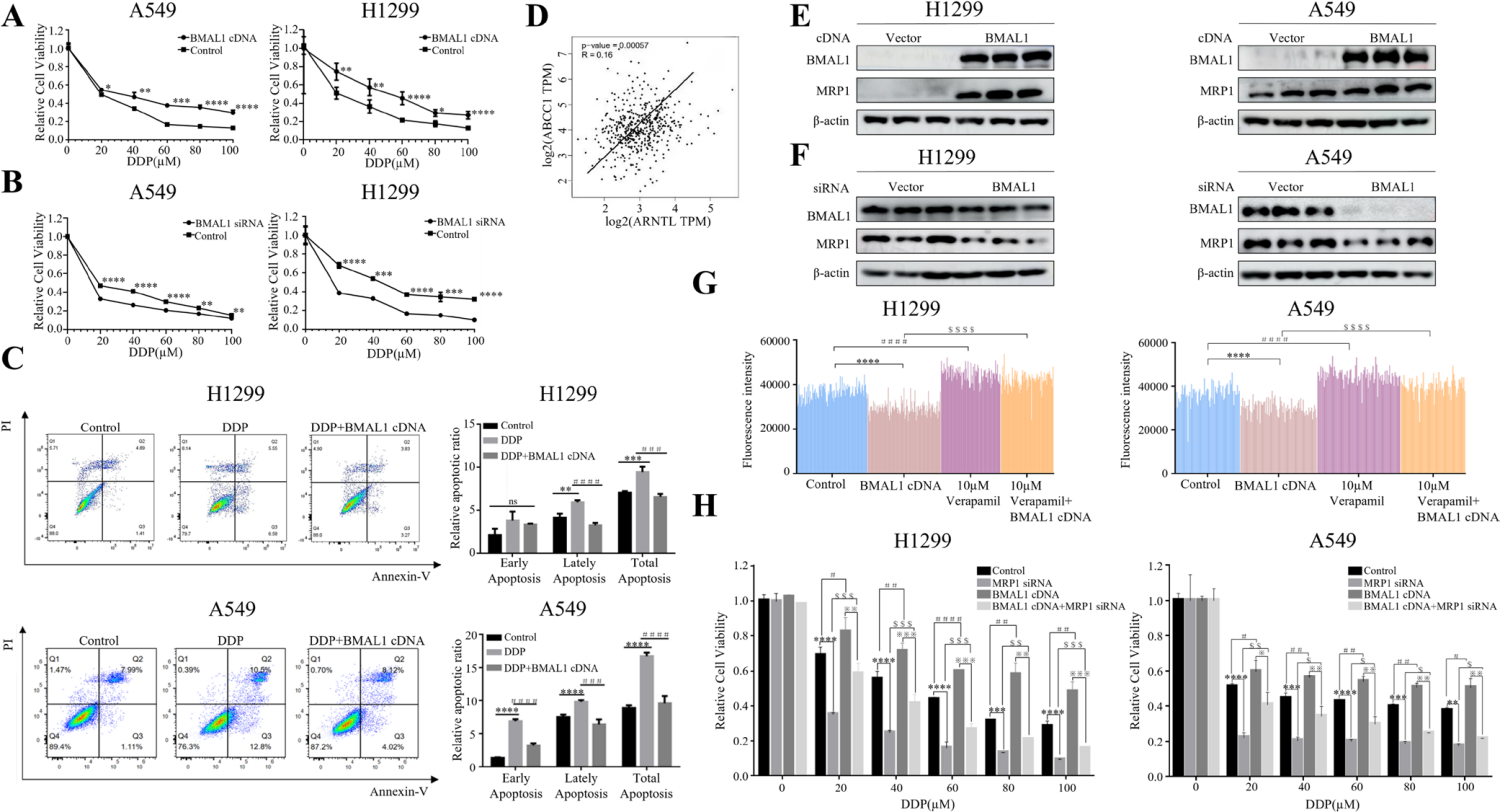
BMAL1 promotes cisplatin resistance in lung cancer cells via MRP1 regulation. (A) A549 and H1299 cells were transfected with BMAL1 cDNA for 48 h, treated with cisplatin (0–100 μM) for 48 h and proliferation was assessed by MTT assay. Data: Mean ± SD (triplicates; *p < 0.05, **p < 0.01, ***p < 0.001, ****p < 0.0001 vs. control; ANOVA with Dunnett's test). (B) Cells transfected with control siRNA or BMAL1 siRNA for 24 h, treated with cisplatin (0–100 μM) for 48 h and analyzed via MTT. Data: Mean ± SD (triplicates; *p < 0.05, **p < 0.01, ***p < 0.001, ****p < 0.0001 vs. control; ANOVA with Dunnett's test). (C) BMAL1 cDNA‐transfected cells treated with cisplatin (25 μM) for 4 h, washed, and cultured for an additional 44 h before flow cytometry analysis. Data: mean ± SD (triplicates; **p < 0.01, ***p < 0.001, ****p < 0.0001 vs. control; ^###^ *p* < 0.001, ^####^ *p* < 0.0001 vs. DDP‐treated cells; NS: not significant; ANOVA with Dunnett’s test). (D) Correlation between BMAL1 (ARNTL) and MRP/ABCC expression in LUAD patients (TCGA database). (E, F) H1299 and A549 cells were transfected with BMAL1 cDNA (E), BMAL1 siRNA (F), or corresponding controls (Vector). BMAL1 and MRP1 protein levels were confirmed by Western blotting. (G) Hoechst 33342 efflux assays in BMAL1 cDNA‐transfected cells in the presence and absence of ABC transporter inhibitor 10 µM Verapamil. Data: mean ± SD (triplicates; ****p < 0.0001 vs. untreated control; ^####^ *p* < 0.0001 vs. Verapamil alone; ^$$$$^ *p* < 0.0001 vs. BMAL1 cDNA alone; ANOVA with Dunnett’s test). (H) Proliferation of cells co‐transfected with BMAL1 cDNA, MRP1 siRNA or both, followed by DDP treatment (0–100 μM). Data: Mean ± SD (triplicates; **p < 0.01, ***p < 0.001, ****p < 0.0001 vs. control; ^##^ *p* < 0.01 vs. BMAL1 cDNA; ^$$^ *p* < 0.01 vs. MRP1 siRNA; ^※※^ *p* < 0.01, ^※※※^ *p* < 0.001 vs. BMAL1 cDNA + MRP1 siRNA; ANOVA with Dunnett’s test).

The DDP concentrations used were based on our and other lab studies of previous IC50 determinations and are within the range reported in other NSCLC resistance studies [13, 14]. Our findings revealed that overexpression of BMAL1 increased the resistance of lung cancer cells to cisplatin (Figure 1A), whereas the knockdown of BMAL1 enhanced their sensitivity to the drug (Figure 1B). These results were further corroborated by apoptosis experiments, which showed similar trends (Figure 1C). Based on these observations, we hypothesized that BMAL1 promotes resistance to chemotherapy drugs in lung cancer cells.

To further explore this mechanism, we conducted bioinformatics analysis and discovered a positive correlation between BMAL1 and the key resistance gene MRP1/ABCC (Figure 1D). Western blot analysis confirmed that overexpression of BMAL1 upregulated MRP1 expression (Figure 1E), while knockdown of BMAL1 reduced MRP1 levels (Figure 1F). Given that MRP1 is known to expel drugs from cells, we investigated whether BMAL1 influenced drug efflux. Indeed, we found that BMAL1 enhanced the efflux of fluorescent dyes from lung cancer cells (Figure 1G). Importantly, knockdown of MRP1 reversed the increased cisplatin resistance induced by BMAL1 overexpression (Figure 1H). These results collectively indicate that MRP1 mediates the cisplatin resistance induced by BMAL1.

### 3.2 BMAL1 Increases Glycolysis‐Dependent MRP1 Expression via HIF‐1α

**FIGURE 2:**
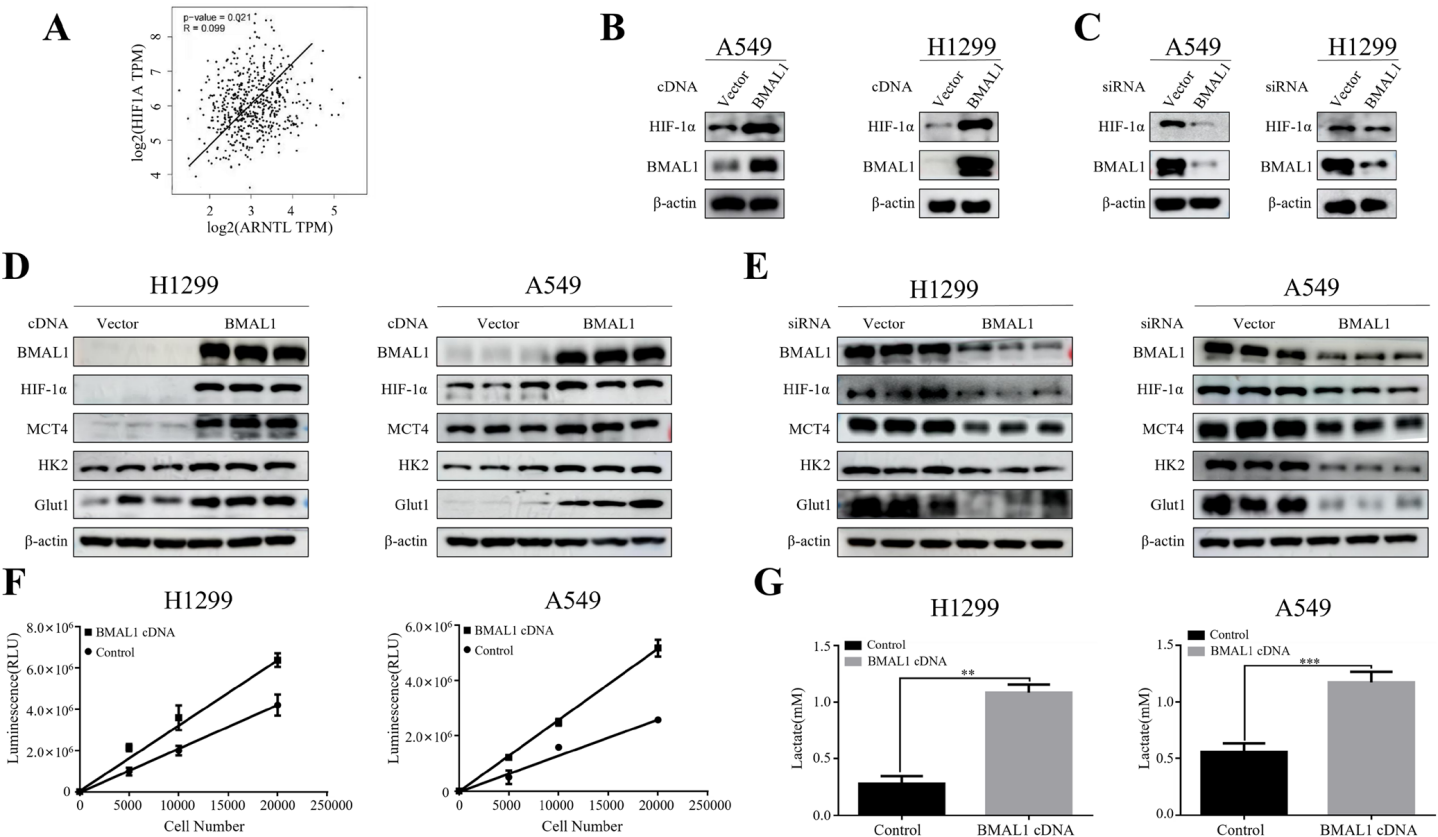
BMAL1 enhances glycolysis‐dependent MRP1 expression via HIF‐1α. (A) Correlation of ARNTL (BMAL1) with HIF‐1α in LUAD (TCGA database). (B, C) HIF‐1α and BMAL1 protein levels in cells transfected with BMAL1 cDNA (B) or siRNA (C), each alongside empty vector controls (Vector) (Western blot). (D, E) BMAL1 cDNA (D) or siRNA (E) effects on HIF‐1α, MCT4, HK2 and Glut1 levels, the expression levels of each protein are referenced by empty vectors (Vector) (Western blot). (F) Glucose uptake in BMAL1‐overexpressing cells at increasing cell densities (5000–20 000 cells). (G) Lactate secretion levels in BMAL1‐overexpressing cells. Data: Mean ± SD (triplicates; **p < 0.01, ***p < 0.001 vs. control; ANOVA with Dunnett's test).

Having established BMAL1's regulation of MRP1, we next investigated whether metabolic reprogramming—a known BMAL1‐associated process—serves as the intermediary mechanism. Although we did not find direct binding sites for BMAL1 in the MRP1 promoter sequence, our previous work demonstrated that metabolic reprogramming in lung cancer cells enhances glycolysis‐induced MRP1 expression [13]. Given the well‐established link between circadian rhythms and cellular metabolism [15], we hypothesized that BMAL1 might influence glycolysis in lung cancer cells. Bioinformatics analysis revealed a positive correlation between BMAL1 and the key tumor metabolism regulator Hif‐1α (Figure 2A). Previous work demonstrated that BMAL1 directly binds to the promoter region of HIF‐1α and control HIF‐1α activity [16]. Peek et al. show that in the skeletal muscle BMAL1 promotes HIF1α—to control oxygenic mitochondrial respiration and anaerobic glycolysis [17]. Consistently, Western blot experiments confirmed that overexpression of BMAL1 upregulated Hif‐1α protein expression (Figure 2B), while knockdown of BMAL1 significantly reduced Hif‐1α levels (Figure 2C). Additionally, we found that BMAL1 overexpression upregulated key glycolysis genes, including HK2, MCT4 and Glut‐1 (Figure 2D), whereas BMAL1 depletion reduced their expression (Figure 2E). Functional assays further demonstrated that BMAL1 overexpression increased glucose transport and lactate production (Figure 2F,G). These findings collectively suggest that BMAL1 promotes glycolysis in lung cancer cells.

### 3.3 BMAL1 Upregulates MRP1 via Lactic Acid Signaling

**FIGURE 3:**
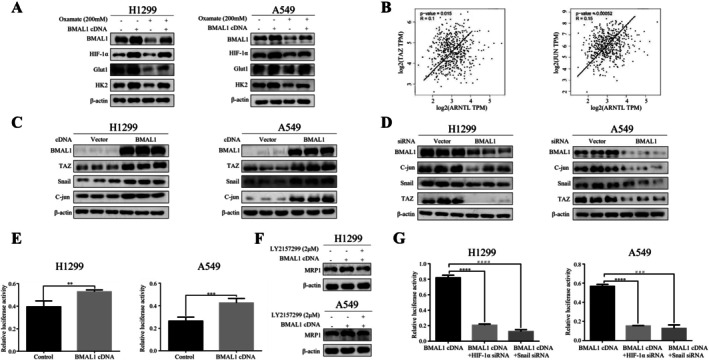
BMAL1 Upregulates MRP1 via Lactic Acid Signaling. (A) BMAL1 cDNA‐transfected cells ± sodium oxamate (200 mM, LDHA inhibitor) were analyzed for BMAL1, HIF‐1α, Glut1 and HK2 (Western blot). (B) Correlation of ARNTL (BMAL1) with TAZ and C‐Jun in LUAD (TCGA). (C, D) Western blot was performed to detect TAZ, Snail and C‐Jun in BMAL1 cDNA‐transfected (C) or siRNA‐transfected (D) cells, with empty vector controls (Vector) included. (E) Luciferase activity of MRP1 promoter in BMAL1‐overexpressing cells. Data: Mean ± SD (triplicates; **p < 0.01, ***p < 0.001 vs. Control; ANOVA with Dunnett's test). (F) Western blot indicated that the robust induction of MRP1 by BMAL1 was blunted by addition of TGF‐βRI specific inhibitor LY2157299. (G) MRP1 promoter activity in BMAL1 cDNA‐transfected cells ± HIF‐1α/Snail siRNA. Data: Mean ± SD (triplicates; ****p < 0.0001 vs. HIF‐1α siRNA; ^###^ *p* < 0.001, ^####^ *p* < 0.0001 vs. Snail siRNA; ANOVA with Dunnett's test).

Our previous work highlighted the role of lactic acid in MRP1 expression induced by metabolic reprogramming. Since BMAL1‐driven glycolysis could elevate lactate, a metabolite also linked to MRP1 expression, we explored whether lactate directly bridges BMAL1 activity to chemoresistance. Western blot analysis confirmed that inhibition of lactic acid by the LDHA inhibitor oxamate reversed the induction of key glycolysis genes by BMAL1 (Figure 3A). Lactic acid is known to mediate MRP1 expression through the transforming growth factor‐beta 1 (TGF‐β1) and Hippo pathways, forming complexes with Snail, TAZ and c‐Jun on the MRP1 promoter [13].

Bioinformatics analysis revealed a positive correlation between BMAL1 and TAZ as well as c‐Jun (Figure 3B). Western blotting confirmed that BMAL1 upregulated Snail, TAZ and c‐Jun (Figure 3C), while suppression of BMAL1 reduced their expression (Figure 3D). Reporter gene assays demonstrated that BMAL1 overexpression significantly increased MRP1 promoter activity (Figure 3E). Moreover, our previous work demonstrated that the activation of the TGF‐β1 pathway by lactate‐derived acidification results in the induction of Snail expression [18]. Therefore, we performed the experiment to verify the BMAL1/TGF‐β1/Snail/MRP axis using the TGF‐β1 inhibitor. We found that the robust induction of MRP1 by BMAL1 was blunted by the addition of the TGF‐βRI specific inhibitor LY2157299 (Figure 3F). Importantly, this BMAL1‐induced MRP1 promoter activity was reversed by Snail and Hif‐1α siRNA (Figure 3G). These results indicate that lactic acid is a key factor mediating BMAL1 regulation of MRP1 expression.

### 3.4 Cisplatin and Etoposide Induce BMAL1 via AKT Signaling

**FIGURE 4:**
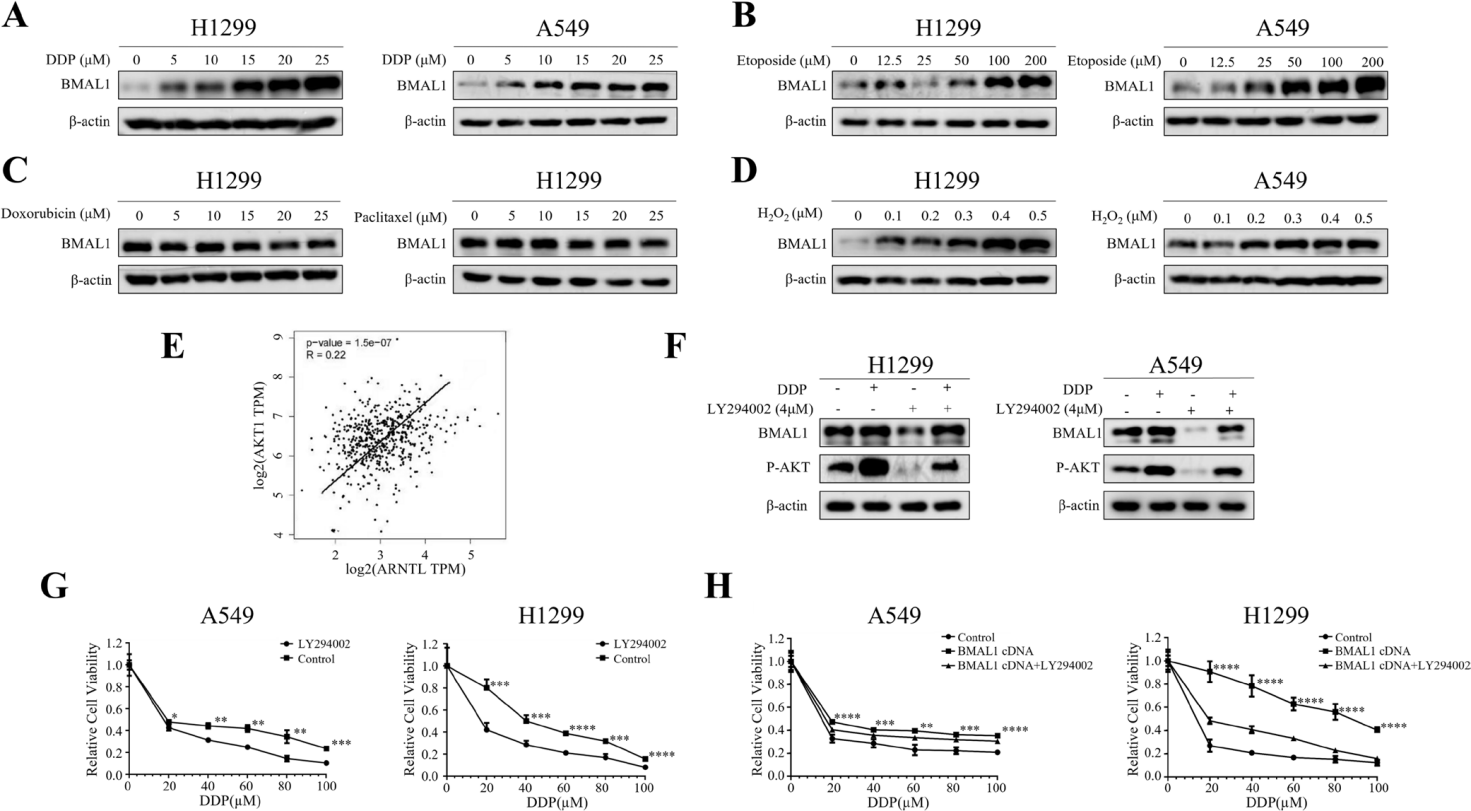
Cisplatin and etoposide induce BMAL1 via AKT signaling. (A–D) BMAL1 protein levels in cells treated with cisplatin (A), etoposide (B), doxorubicin/paclitaxel (C), or H_2_O_2_ (D) for 4 h (Western blot). (E) Correlation of ARNTL (BMAL1) with AKT in LUAD (TCGA). (F) BMAL1 and phospho‐AKT (p‐AKT) levels in cisplatin‐treated (25 μM, 4 h) cells ± LY294002 (4 μM, AKT inhibitor) (Western blot). (G, H) Proliferation of cisplatin‐treated (0–100 μM, 48 h) cells ± LY294002 (G) or LY294002 + BMAL1 cDNA (H). Data: Mean ± SD (triplicates; *p < 0.05, **p < 0.01, ***p < 0.001, ****p < 0.0001 vs. Control; ANOVA with Dunnett's test).

Having delineated BMAL1's downstream effects, we asked whether chemotherapy itself influences BMAL1 expression, potentially creating a resistance feedback loop. We found that cisplatin upregulated BMAL1 in a dose‐dependent manner (Figure 4A). Similarly, the chemotherapeutic drug etoposide also dose‐dependently upregulated BMAL1 (Figure 4B). Interestingly, BMAL1 expression was not altered by DOX and PTX treatment in H1299 cells (Figure 4C).

In addition to causing DNA damage, cisplatin and etoposide are known to induce oxidative stress in cells [13, 19]. We observed that hydrogen peroxide treatment upregulated BMAL1 in a dose‐dependent manner (Figure 4D). Given that oxidative stress stimulates the AKT signaling pathway, we explored the relationship between BMAL1 and AKT. Bioinformatics analysis revealed a positive correlation between BMAL1 and AKT (Figure 4E). We confirmed that cisplatin activates AKT and that AKT inhibition with LY294002 reversed cisplatin‐induced BMAL1 upregulation (Figure 4F). Notably, LY294002 not only increased cell death induced by cisplatin (Figure 4G) but also reversed BMAL1‐enhanced cell viability (Figure 4H). These findings suggest that cisplatin‐induced oxidative damage upregulates BMAL1 through AKT signaling.

## 4 Discussion

The poor prognosis of advanced lung cancer is often attributed to robust resistance to chemotherapy. Our study identifies BMAL1, a core circadian rhythm gene, as a critical driver of cisplatin resistance in lung cancer cells by upregulating the multidrug resistance protein MRP1 through metabolic reprogramming and oxidative stress pathways. These findings advance our understanding of chemoresistance mechanisms and highlight BMAL1 as a potential therapeutic target.

Previous work implies a broad pro‐tumorigenic function of BMAL1 [20], a direct and mechanistic link between BMAL1 and drug chemoresistance has remained less explored and is the central novelty of our work. While DNA repair is a known BMAL1 function through directly interacting with repair proteins to facilitate double‐strand break repair [21], its involvement in drug resistance via modulation of drug efflux machinery remains unaddressed. We specifically link its circadian oscillation to the rhythmic activation of a specific drug efflux pump MRP1. We show that BMAL1's peak expression directly drives the transcription of this pump, creating predictable windows of drug tolerance that are mechanistically distinct from its role in repairing DNA damage induced by radiation or other agents. This study fills this gap by investigating BMAL1 as a novel regulator of chemoresistance in NSCLC.

Our results demonstrate that BMAL1 enhances glycolysis in lung cancer cells, increasing lactate production, which in turn activates the TAZ/c‐Jun/Snail complex to upregulate MRP1. This is consistent with prior studies showing that metabolic shifts, particularly glycolysis, promote drug efflux via MRP1 in NSCLC [13]. Importantly, BMAL1's regulation of MRP1 appears indirect, mediated through its control of Hif‐1α and glycolytic enzymes. This mechanism is distinct from canonical circadian gene regulation, as BMAL1 does not directly bind the MRP1 promoter. Instead, our findings suggest BMAL1 acts as a metabolic orchestrator, linking circadian disruption to chemoresistance.

Notably, recent work by Ma et al. found that NPAS2, another core circadian gene, promotes aerobic glycolysis in prostate cancer via HIF‐1α [16]. NPAS2 is shown to be upregulated in PCa tissues and cell lines, promoting cell survival and tumor growth by enhancing aerobic glycolysis and inhibiting oxidative phosphorylation. This work revealed NPAS2 increases the expression of HIF‐1α, a key regulator of glycolysis and upregulates glycolytic genes such as HK2, PKM2, GLUT1, and MCT4. This metabolic reprogramming supports tumor progression. The significance of HIF‐1α as a key player in connecting circadian regulation to metabolic alterations in cancer cells is consistent with our work and this study. These findings also highlight the intricate interplay between circadian regulation, metabolic reprogramming and tumor progression, offering novel insights into potential therapeutic strategies.

This study is also consistent with the growing body of evidence that metabolic reprogramming, particularly the Warburg effect, plays a critical role in chemoresistance [22]. This finding also reveals that chemotherapy drugs like cisplatin and etoposide induce BMAL1 expression through AKT signaling in response to oxidative stress, creating a self‐reinforcing resistance mechanism. This is in line with previous research showing that oxidative stress and metabolic shifts can activate adaptive resistance pathways in cancer cells [13]. However, the study diverges from findings in other cancers, such as colorectal cancer, where BMAL1 promotes cell migration and invasion via the ERK‐ and JNK‐dependent upregulation of c‐Myc [20], a well‐known oncogene involved in epithelial‐to‐mesenchymal transition and tumor progression. This suggests that the role of BMAL1 in cancer progression and resistance may be context‐dependent, influenced by the specific metabolic and molecular landscape of the tumor.

The role of BMAL1 in chemoresistance extends beyond metabolism. We show that cisplatin and etoposide upregulate BMAL1 via AKT activation in response to oxidative stress, which may allow cancer cells to counteract drug‐induced DNA damage. This is in line with reports that BMAL1 enhances DNA repair by promoting double‐strand break repair [21]. However, BMAL1's effects are drug‐specific: it does not confer resistance to DOX or PTX, likely due to differences in their mechanisms of action. Such specificity underscores the need for precision in targeting BMAL1‐dependent pathways.

Our data provides a strong mechanistic rationale for timing chemotherapy administration to coincide with the trough of BMAL1 expression (and thus low efflux pump activity), which could significantly enhance drug accumulation and efficacy while reducing side effects. We also propose that BMAL1 expression (measured via immunohistochemistry or liquid biopsy for circulating tumor RNA) could be used to stratify patients: those with low BMAL1 may benefit from standard chemotherapy, while those with high BMAL1 may require combinatorial therapies. For patients with severe BMAL1 upregulation, combining BMAL1 inhibitors with chemotherapeutic drugs could synergistically reverse resistance.

In conclusion, the research presented in these documents advances our understanding of the complex interplay between circadian rhythms, metabolism and cancer progression. While BMAL1 emerges as a critical regulator of chemoresistance in both CRC and NSCLC, its role appears to be highly context‐specific, influenced by the tumor type, metabolic state and TME. Future studies should validate these findings in established resistance models and patient‐derived xenograft (PDX) models to develop more effective therapeutic strategies. The findings also highlight the potential of chronotherapy—timing chemotherapy to coincide with BMAL1's circadian trough—as a promising approach to maximize therapeutic efficacy and minimize resistance. Overall, these studies provide a roadmap for targeting circadian‐metabolic crosstalk to improve outcomes in cancer patients with limited treatment options.

## 5 Conclusion

Lung cancer claims millions of lives annually, with over 60% of NSCLC patients lacking targetable mutations and only 20% responding to immunotherapy. Chemotherapy remains a cornerstone, yet resistance severely limits its efficacy. While metabolic shifts like the Warburg effect are hallmarks of cancer, their role in chemoresistance—particularly crosstalk with circadian pathways—is poorly defined. BMAL1, a circadian gene, is implicated in tumor metabolism, but its mechanistic role in resistance is less defined. This gap hinders the development of strategies to counteract adaptive resistance triggered by chemotherapy itself. By elucidating how BMAL1 integrates metabolic reprogramming, lactate signaling and oxidative stress to upregulate MRP1, this research addresses a critical unmet need: disrupting the self‐perpetuating circuits that render chemotherapy ineffective. The findings provide a roadmap for overcoming resistance through circadian‐targeted therapies, offering hope for NSCLC patients with limited treatment options.

## Author Contributions

Z.S., Z.Q., C.C., X.T., Y.H., T.X. performed the in vitro experiments. H.W. and X.T. coordinated in all experiments. Z.W. and H.W. designed this study and the experiments, and wrote the manuscript. All authors read and approved the final manuscript.

## Funding

This work was supported by National Natural Science Foundation of China (81872371), Program for Excellent Sci‐tech Innovation Teams of Universities in Anhui Province (2023AH010073), Provincial‐level Quality Project in Higher Education Institutions of Anhui Province (2022jyxm1710), National College Students Innovation and Entrepreneurship Training Program (202110368065, 202310368018, 202510368009), Anhui Provincial Key Science Foundation for Outstanding Young Talent (23AH030108), Health Science Research Project of Anhui Province (AHWJ2022a028), 2023 Annual Key Project Research Fund of Wannan Medical College (WK2023ZZD13), Natural Science Foundation of the Higher Education Institutions of Anhui Province (2025AHGXZK30247).

## Ethics Statement

The manuscript does not include data or description of human participants or animal subjects; therefore, ethics committee approval is not required at that institution.

## Conflicts of Interest

The authors declare no conflicts of interest.

## Data Availability

The data that support the findings of this study are available from the corresponding author upon reasonable request.
